# Arterial Embolization versus Robotic Partial Nephrectomy for the Treatment of Renal Angiomyolipomas

**DOI:** 10.1590/S1677-5538.IBJU.2025.0163

**Published:** 2025-06-10

**Authors:** Idir Ouzaid, Pierre-Etienne Gabriel, Byron Lee, Gauthier Delporte, Philippe Puech, Laurent Lemaitre, Evanguelos Xylinas, Arnauld Villers, Georges-Pascal Haber

**Affiliations:** 1 Paris University Bichat Claude Bernard Hospital Department of Urology Paris France Department of Urology, Bichat Claude Bernard Hospital, Paris University, Paris, France; 2 Glickman Urological and Kidney Institute Ohio USA Glickman Urological and Kidney Institute, Cleveland Clinic, Ohio; 3 CHU de Lille Department of Urology Lille France Department of Urology, CHU de Lille, Lille, France; 4 CHU de Lille Department of Radiology Lille France Department of Radiology, CHU de Lille, Lille, France

**Keywords:** Angiomyolipoma, Robotics, Embolization, Therapeutic

## Abstract

**Objective:**

To compare the outcomes of robotic-assisted partial nephrectomy (RALPN) and selective arterial embolization (SAE) for the treatment of sporadic renal angiomyolipoma (AML).

**Patients and methods:**

The outcomes of patients who were managed by RALPN (n = 191) or SAE (n = 51) for sporadic renal AML were matched (2:1) using a propensity score for analyses. The primary endpoint was therapeutic success defined as the absence of secondary treatment. Secondary endpoints were post-operative complications and renal function preservation (loss of eGFR at 6 months). Univariate and multivariate logistic regression analyses were used to predict factors associated with re-intervention.

**Results:**

Patients baseline characteristics in the matched population (RALP, n=96 vs. SAE, n=48) were balanced. LOS was shorter (mean: 4.2 vs. 3.1 days; p = 0.004) and EBL was lower (327 mL vs. 0 mL, p < 0.0001) in the SAE group. Overall (PN: 15.2% vs. AES: 11.7% p = 0.09) and Clavien-Dindo stratified (p = 0.62) complications were similar in both groups. After a comparable mean follow-up time (33 vs. 40 months, p = 0.63), there was an overall mean loss of eGFR of 7.7±26 mL/min/1.73m2 (p = 0.001). This loss was similar between the two groups (PN: 6.87±26 vs. AES: 11.56±23, p = 0.36). After adjusting for identified confounding factors including tumor size, type of primary intervention (RALPN vs SAE) was the only predictive factor for secondary intervention.

**Conclusion:**

RALPN was associated with decreased need for secondary treatment with no increase in morbidity compared with SAE.

## INTRODUCTION

Renal angiomyolipomas (AML) are benign tumors of the kidney that account for approximately 3% of all renal tumors (1, 2). Smooth muscle, aneurismal vessels, and adipose tissue are three pathological components that define AML (3). Eighty percent of AML are sporadic and 20% are associated with a genetic syndrome such as tuberous sclerosis complex (4). Although most AML are detected incidentally, patients can present with flank pain, recurrent gross hematuria, and life-threatening retroperitoneal hemorrhage (5).

Active surveillance (AS) is the management choice for most asymptomatic patients with AML, but over half of patients will ultimately undergo active treatment (6, 7). For most patients, nephron sparing approaches such as partial nephrectomy (PN) and selective arterial embolization (SAE) are the most used options (8, 9).

Historically, SAE was associated with fewer post-procedural complications and improved preservation of renal function compared with PN. However, SAE also required more secondary procedures compared with PN (10). Recent advances in robot-assisted PN (RALPN) technique have resulted in improved peri-operative outcomes compared with historical series (11, 12).

Thus, our study aimed to examine the outcomes of SAE and RALPN in the management of a contemporary cohort of patients with sporadic renal AML.

## PATIENTS AND METHODS

### Patients and study design

A multi-institutional (n=3), institutional review board (IRB-20-836)-approved database was queried to identify patients who were diagnosed with a sporadic renal AML and primarily treated with RALPN (n = 191) or SAE (n = 51). Diagnosis of AML required a clearly identifiable fat component on CT (13). Patients diagnosed with Tuberous Sclerosis Complex (TSC) syndrome and concomitant or past contralateral kidney disease (cyst, RCC) were excluded from the analyses. AML presented with life-threatening and/or active hemorrhage were excluded from the analysis. Given the retrospective aspect of the study design, individual informed consent was waived.

Baseline characteristics including age, gender, tumor size, and chronic kidney disease (CKD) stage were retrieved. Operative complications, estimated blood loss, length of stay, 90-d postoperative complications according to the Clavien-Dindo classification were also recorded. During follow-up, secondary procedures were triggered upon physician opinion according to patient's symptoms and tumor evolution.

### Hypotheses and endpoints

We hypothesize that RALPN is associated with a decreased need for secondary treatment, without an increase in morbidity compared to SAE.

The primary endpoint was therapeutic success defined as the absence of secondary procedures for the same AML. Secondary endpoints were postoperative complications and estimated glomerular filtration rate (eGFR) 6 months after the intervention.

## Statistical analysis

Differences between patients undergoing RALPN and those undergoing SAE were compared using the Chi-square or Fisher exact tests for categorical variables (presented as proportions) and student t-test or Wilcoxon rank sum test for continuous variables (presented as mean ± standard deviation, SD). Univariate and a multivariate cox regression model were used to predict risk factors for re-intervention.

Since patients were not randomly assigned to either surgical approach, treatment effect estimates are biased if selection biases were left unadjusted. Propensity score and matching techniques have been used to remove bias of measured variables and optimize unbiased estimate of treatment effects. Maximum balance between multivariate covariates were achieved at baseline where a genetic search algorithm was used to determine the optimal weight of each covariate (14). A 2:1 matching method was used with no replacement. Using this algorithm, we were able to match 48 patients who underwent SAE to 96 unique control patients (RALPN group). Statistical analysis was performed using R 3.0.0 (www.r-project.org) and p-values were two-sided and statistical significance was defined as a p<0.05.

## RESULTS

### Baseline characteristics of the patients included in the study

Baseline characteristics of the treatment groups are summarized in Table-1. Patients who underwent SAE were younger (40 vs 52 years, p < 0.0001) and were more likely to have multiple (37% vs 15%, p < 0.001), larger (6.4 vs 4.6 cm, p < 0.0001), and more symptomatic (flank pain: 41% vs. 14%, p < 0.001) tumors.

**Table 1:** Baseline characteristics of the study population before and after matching.

		Before propensity score			After propensity score		
		RALPN	SAE	p	RALPN	SAE	p
		n = 191	n = 52		n = 96	n = 48	
Age, years, mean±SD		52±14	40±14	<0.0001	42±5	41±6	0.28
Gender, n (%)							
	Female	148 (78%)	45 (88%)	0.09	78 (81%)	43 (89%)	0.19
	Male	43 (22%)	6 (12%)		18 (19%)	5 (11%)	
BMI, kg/m^2^, mean±SD		28 ± 8	25 ± 6	0.515	27 ± 6	25 ± 4	0.38
Side, n (%)							
	Right	90 (47%)	20 (38%)	0.7354	44 (46%)	20 (42%)	0.2
	Left	97 (51%)	24 (46%)		50 (52%)	24 (50%)	
	Bilateral	4 (2%)	8 (16%)		2 (2%)	4 (8%)	
Tumor size, cm, mean±SD		4.6 ± 4.1	6.4 ± 3.2	<0.006	5.3 ± 3.1	5.9 ± 2.9	0.26
Symptoms, n (%)							
	Incidentalomas	114 (59%)	14 (27%)	<0.01	42 (51%)	15 (31%)	0.48
	Flank pain	36 (19%)	25 (49%)		36 (37%)	24 (50%)	
	Hematuria	26 (14%)	6 (12%)		10 (10%)	5 (10%)	
	Retroperitoneal hematoma	15 (8%)	6 (12%)		8 (10%)	4 (9%)	
Number of tumors, n (%)							
	1	131 (68%)	24 (46%)	<0.001	49 (68%)	22 (46%)	0.79
	*2*	21 (11%)	9 (17%)		18 (11%)	9 (17%)	
	Multiples	29 (15%)	19 (37%)		29 (15%)	17 (37%)	
eGFR, mL/min/1.73m^2^, mean ± SD		92 ± 27	100 ± 37	0.098	92 ± 24	96 ± 2.9	0.032

RALPN = Robot-assisted laparoscopic partial nephrectomy; SAE = Selective arterial embolization; eGFR = estimated glomerular filtration rate

The propensity score matching resulted in 48 (SAE) and 96 (RALPN) patients’ groups with similar baseline characteristics allowing the comparison of their respective outcomes (Table-1).

### Perioperative outcomes according to the type of treatment in the matched population

Patients who underwent SAE had shorter hospital stay (3.1 days vs 4.2 days, p = 0.006) compared with patients who underwent RALPN (Table-2). SAE was also associated with lower blood loss than RALPN. However, overall complications rates were not significantly different between RALPN and SAE (15.2% vs. 11.7%, p = 0.09). Clavien-Dindo minor (Grade 1-2) and major (Grade 3-4) complication rates in the two groups and did not find any statistically significant difference as well (p = 0.62).

**Table 2:** Perioperative outcomes of RALPN and SAE in the treatment of renal AML.

		RALPN	SAE	p
		n = 96	n = 48	
Hospital stays, days, mean± SD		4.19 ± 1.65	3.14 ± 3.49	**0.006**
Blood loss, mL, mean± SD		327 ± 436	-	**<0.0001**
Overall complications, n (%)		14 (14.5%)	6 (12.5%)	0.09
**Postoperative complications, n (%)**				
	Minor (1-2)	10 (10.4%)	4 (8.3%)	0.62
	Major (3-5)	4 (4.1%)	2 (4.1%)	
Postoperative eGFR, mL/min/1,73m^2^, mean±SD		84 ± 30	87 ± 30	0.06

RALPN = Robot-assisted laparoscopic partial nephrectomy; SAE = Selective arterial embolization; eGFR = estimated glomerular filtration rate

### Reintervention outcomes in the matched population

After median follow-up of 33 months (IQR: 6-229) and 40 months (IQR: 6-173) (p=0.63), 4 (4.1%) and 14 (29.1%) patients underwent a secondary treatment in the RALPN and SAE group, respectively. Secondary treatments in the RALPN group included repeat PN (n=2) radical nephrectomy (n=1), and cryoablation (n=1) for tumor recurrence. Mean tumor size in the failed RALPN group was 6.5 cm (range: 4,5-7.7 cm). In the SAE group, secondary treatments included repeat SAE in (n=12) and RALPN (n=2). Mean tumor size in the failed SAE group was 6 cm (range: 4-8).

### Renal function preservation in the matched population

During follow-up, overall mean loss of eGFR was 7.7±26 mL/min/1.73m^2^ after any intervention. This loss was similar between the two groups (RALPN: 6.87±26 vs. SAE: 11.56±23, p = 0.36).

### Predictors of reintervention in the matched population

In the univariable analysis, patient age, tumor size, tumor number, and treatment type were associated with secondary treatment. However, after adjusting for confounding factors, the multivariable logistic regression model showed that the primary treatment type (RALPN versus SAE) was the only significant factor associated with secondary treatment (Table-3). Patients primarily treated with SAE were more likely to undergo a secondary procedure during follow-up (RALPN versus SAE: HR :0.16; 95%CI: 0.05-0.55; p = 0.003).

**Table 3:** Univariate and multivariate analyses exploring predictive factors of reintervention.

Variables	Univariate analyses		Multivariate analyses	
	HR (CI 95%)	p	HR (CI 95%)	p
**Age**	0.98 (0.93-0.98)	0.005	0.98 (0.95-1.02)	0.405
**Tumor size**	1.10 (1.01-1.19)	0.02	1.04 (0.93-1.16)	0.404
**Symptoms** **(Yes vs. No)**	1.09 (0.87-4.45)	0.13	1.76 (0.61-5.05)	0.550
**Number of tumors (multiple vs. unique)**	4.04 (1.70-9.63)	0.002	1.70 (0.62-4.63)	0.291
**Treatment type** **(RALPN vs. SAE)**	0.11 (0.04-0.29)	<0.0001	0.16 (0.05-0.55)	0.003

RALPN = Robot-assisted laparoscopic partial nephrectomy; SAE = Selective arterial embolization

## DISCUSSION

Given the benign nature of this disease, AS is the management of choice for AML. However, symptomatic AML may necessitate active treatment, especially in the case of life-threatening retroperitoneal hemorrhage (15, 16). Surgery and SAE are used in respectively in 31% and 17% of the cases (16). Contemporary studies comparing peri-procedural, tumor control, and functional outcomes between surgery and SAE in AML management are lacking, with all studies published more than a decade ago (17–19). Since then, there have been significant advances in both interventional radiology and surgical techniques. Herein, we report a retrospective study comparing RALPN and SAE. To the best of our knowledge, this is the first and largest series to be specifically designed for this purpose in the modern era of robotic assisted surgery.

Our findings suggest the superiority of the surgical approach with respect to tumor control. Over a comparable follow-up period, SAE was associated with a failure rate of 29.1% while less than 5% of patients undergoing RALPN required repeat intervention. These findings are consistent with historical comparative studies examining effectiveness of SAE and RALPN in tumor control (17–19) . Peri-procedural complications were rare in both modalities; and when they did occur, most were minor (Clavien-Dindo 1-2). Importantly, renal function was well-preserved, and there was no significant difference in post-procedural eGFR between SAE and RALPN (10).

The management of sporadic renal AML depends on clinical presentation, and there is a role for both SAE and RALPN. The main criteria that drive decision making are the presence of major or life-threatening symptoms and radiologic characteristics (size, location, number). For example, SAE is often used in cases with retroperitoneal hemorrhage, multiple, or large tumors. In these cases, SAE offers a relatively rapid and safe approach for temporization in an urgent setting, but it is important to note that even after an ablative procedure, RALPN should be considered a viable definitive treatment (20). In fact, secondary bleeding or tumor growth occurred more frequently in the follow up period, which necessitated definitive treatment either with repeat SAE or surgery. On the other hand, our data suggest that RALPN would be preferred in situations where lack of immediacy does not lead to adverse clinical outcomes. We showed that modern RALPN technique is safe, effective, and resulted in good preservation of renal function (21).

**Figure 1:**
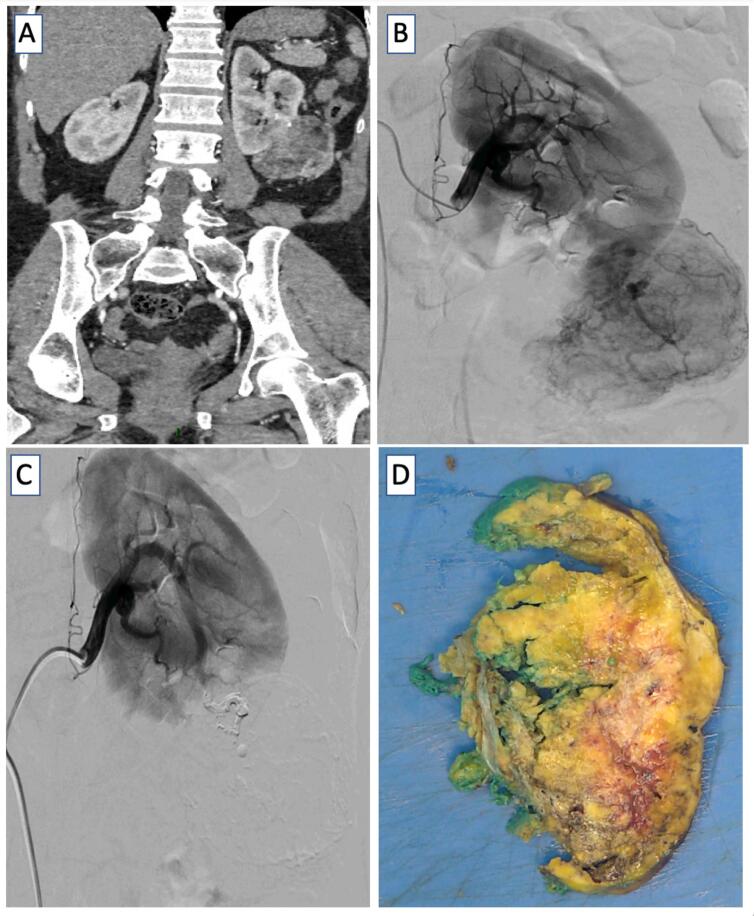
Combined approach for a 8 cm symptomatic AML.

The diagnosis of renal AML is based on axial imaging and uncomplicated in most cases. Sometimes, fat-poor AMLs are encountered, which may warrant further investigation (e.g. renal mass biopsy) to rule out renal cell carcinoma or epithelioid AML that may require extirpation (22). Nonetheless, most AML can be managed with active surveillance, and the traditional 4-cm cut-off alone should not trigger active treatment (16, 23). During active surveillance, changes in tumor growth kinetics, symptoms, and patient preference can serve as triggers for intervention. For those who require treatment, the presence of life-threatening hemorrhage should be managed with SAE before definitive treatment with excision. In the absence of the active bleeding, and given the failure rate up to 30% of the cases, we recommend upfront nephron sparing surgery (NSS) (15). During surgical planning, if unacceptable blood loss and/or prolonged warm ischemia time (>25-30 min) are anticipated, one option would be to employ a combined approach with pre-operative SAE followed by RALPN. This approach minimizes warm ischemia time, allows maximum parenchymal preservation, and decreases need for repeat intervention (Figure-1) (24).

The CT scan (A) shows a distinct fat component (-63 HU). Pre-embolization (B) and post-embolization angiography (C) before off-clamp robotic assisted laparoscopic partial nephrectomy with an estimated blood loss <100mL. Pathology revealed a typical renal AML staining positive for HMB45 (Human Antibody 45), Melan A (Melanoma A), and MSA (Muscle Specific Actin).

Our study is limited by its retrospective design and resulting selection bias. Specifically, SAE was more frequently used than RALPN in symptomatic patients and those with larger tumors. To minimize this bias, we adjusted our analyses for all available and measured confounding factors, and differences using a propensity score strategies. Secondly, the study period in the SAE group spanned longer than the RALPN group, and earlier SAE procedures may have benefited from advances in embolization agents and stenting materials with potential better outcomes (25).

In summary, our findings suggest, based on a large propensity score study, the superiority of RALPN in achieving tumor control and renal preservation with acceptable perioperative morbidity. RALPN is preferred in scenarios where immediate treatment (ie : active bleeding) is not needed to avoid an adverse clinical outcome.

## CONCLUSIONS

This study compares modern robotic-assisted partial nephrectomy to selective arterial embolization in the management of sporadic renal angiomyolipoma. Robotic-assisted partial nephrectomy was associated with less treatment failure than selective arterial embolization with similar preservation in renal function. Moreover, advances in robotic surgery have decreased peri-operative morbidity and complication rates. Unless the patient has a life-threatening hemorrhage, they should be advised to consider robotic-assisted partial nephrectomy as a definitive treatment for sporadic renal angiomyolipoma.
